# Neuroprem 2: An Italian Study of Neurodevelopmental Outcomes of Very Low Birth Weight Infants

**DOI:** 10.3389/fped.2021.697100

**Published:** 2021-09-13

**Authors:** Licia Lugli, Luca Bedetti, Isotta Guidotti, Marisa Pugliese, Odoardo Picciolini, Maria Federica Roversi, Elisa DellaCasa Muttini, Laura Lucaccioni, Natascia Bertoncelli, Gina Ancora, Giancarlo Gargano, Fabio Mosca, Fabrizio Sandri, Luigi Tommaso Corvaglia, Agostina Solinas, Serafina Perrone, Marcello Stella, The Neuroprem Working Group, Antonella Luglio, Lorenzo Iughetti, Alberto Berardi, Fabrizio Ferrari

**Affiliations:** ^1^Neonatal Intensive Care Unit, University Hospital of Modena, Modena, Italy; ^2^PhD Program in Clinical and Experimental Medicine, University of Modena and Reggio Emilia, Modena, Italy; ^3^Pediatric Unit, Department of Medical and Surgical Sciences of Mothers, Children and Adults, University of Modena and Reggio Emilia, Modena, Italy; ^4^Psychology Unit, University Hospital of Modena and Reggio Emilia, Modena, Italy; ^5^Physical and Rehabilitation Medicine, Fondazione IRCCS Ca' Granda Ospedale Maggiore Policlinico, University of Milan, Milan, Italy; ^6^Neonatal Intensive Care Unit, Infermi Hospital of Rimini, Rimini, Italy; ^7^Neonatal Intensive Care Unit, Azienda Unità Sanitaria Locale-IRCCS, Reggio Emilia, Italy; ^8^Neonatal Intensive Care Unit, Fondazione IRCCS Ca' Granda Ospedale Maggiore Policlinico, University of Milan, Milan, Italy; ^9^Neonatal Intensive Care Unit, Maggiore Hospital of Bologna, Bologna, Italy; ^10^Neonatal Intensive Care Unit, Sant'Orsola Malpighi University Hospital of Bologna, Bologna, Italy; ^11^Neonatal Intensive Care Unit, Sant'Anna Hospital of Ferrara, Ferrara, Italy; ^12^Neonatal Intensive Care Unit, University Hospital of Parma, Parma, Italy; ^13^Neonatal Intensive Care Unit, Bufalini Hospital of Cesena, Cesena, Italy; ^14^Department of Medical and Surgical Sciences of Mothers, Children and Adults, Pediatric Postgraduate School, University of Modena and Reggio Emilia, Modena, Italy

**Keywords:** very low birth weight, preterm, neuro-developmental outcome, follow-up, network

## Abstract

**Background:** Despite the increased survival of preterm newborns worldwide, the risk of neurodevelopmental disabilities remains high. Analyzing the outcomes of the preterm population can identify risk factors and enable specific early interventions.

**Aims:** Neuroprem is a prospective cohort study of very low birth weight (VLBW) infants that aims to evaluate the neurodevelopmental outcomes and risk factors for severe functional disability at 2 years of corrected age.

**Methods:** Nine Italian neonatal intensive care units participated in the network. The Griffiths Mental Developmental Scales (GMDS-R) or the Bayley Scales of Infant and Toddler Development (BSDI III) and a neuro-functional evaluation (according to the International Classification of Disability and Health and Neuro-Functional Assessment, or NFA ICF-CY) were administered to VLBW infants at 24 months of corrected age. The primary outcome measure was severe functional disability, defined as cerebral palsy, bilateral blindness, deafness, an NFA ICF-CY of >2, a BSDI III cognitive composite score of <2 SD, or a GMDS-R global quotient score of <2 SD. Perinatal risk factors for severe functional disability were assessed through multivariate logistic regression analysis.

**Results:** Among 502 VLBW survivors who completed the 24-month follow-up, 48 (9.6%) presented severe functional disability, of whom 27 had cerebral palsy (5.4%). Rates of severe functional disability and cerebral palsy were higher in neonates with a lower gestational age (*p* < 0.001). Overall, 147 infants (29.3%) were referred to neuromotor intervention. In the multivariate regression model, gestational age at birth OR 0.79; 95% CI 0.67–0.90; *p* = 0.001) and periventricular-intraventricular hemorrhage (OR 2.51; 95% CI 1.19–5.26; *p* = 0.015) were significantly associated with severe functional disability.

**Conclusion:** Neuroprem 2 provides updated information on the neurodevelopmental outcomes of VLBW infants in a large Italian cohort. The overall rate of neurodevelopmental disabilities was quite lower than reported in the previous literature. These data indicate the need for structured follow-up programs from a national neonatal network perspective.

## Background

Prematurity is a major global health problem and the leading cause of death in children under 5 years old. Over the past few decades, advances in the clinical care of preterm infants have led to improved survival in the neonatal age group and beyond. Thus, more premature infants survive with neurodevelopmental morbidities of variable severity, often resulting in lifelong disability. The high rates of neurological and developmental problems reported in survivors are concerning to both professionals and the public. Among extremely preterm neonates (22–26 weeks gestation), survival without neurodevelopmental impairment at 2 years of age ranges from 20 to 42% (1–3). Indeed, in absolute numbers, infants born very or moderately preterm represent a large proportion of preterm births, accounting for most children with motor, cognitive, or behavioral deficits and learning disabilities (4–6). Gestational age (GA), birth weight, sex, multiple birth, antenatal corticosteroid administration, neonatal infection, necrotizing enterocolitis (NEC), and major brain lesions, such as periventricular leukomalacia (PVL) and intraventricular hemorrhage (IVH), have been shown to influence both short- and long-term outcomes (7–9). Analyzing outcomes in the preterm population may identify risk factors that could potentially be targeted by specific early intervention (10–12). In addition, decisions on the provision of intensive vs. palliative care and on the counseling of parents of extremely preterm infants are based on the expected incidence of mortality and poor long-term outcome. Therefore, it is of paramount importance that recent, representative outcome data be available. Up-to-date data are scant regarding the neurodevelopment of very low birth weight (VLBW) infants, and national networks on preterm neurological outcomes are still lacking in Italy, but the Neuroprem study has recently produced preliminary data on the neurodevelopmental outcomes of a VLBW cohort in an Italian region during a 1-year period (13). Neuroprem is now expanding to a larger Italian area including nine neonatal intensive care units NICUs and to a longer study period (Neuroprem 2). Herein, we report on a multicenter prospective cohort study of VLBW preterm infants, which evaluated the neurodevelopmental outcomes and risk factors for severe functional disability at 2 years of corrected age.

## Methods

### Study Design

Neuroprem is an Italian network that assesses VLBW neurodevelopmental outcomes. Nine tertiary level NICUs in two Italian regions, all participating in the Vermont Oxford Network Database (VON) (14), joined this prospective cohort study. Before starting patient enrollment, the NICUs participated in seminars and meetings to define and share the study protocol. Anonymized data, including perinatal and neurodevelopmental follow-up data, were collected in a common data collection format through a web platform. The study enrolled VLBW infants born from 1 January 2016 through 31 December 2018 (and included in VON) and collected their neurodevelopmental data at 24 months (corrected for prematurity). Genetic abnormalities or major malformations were excluded. Several perinatal factors in the VON were evaluated, including birth weight, GA, site and mode of delivery, ethnicity, gender, multiple gestation, prenatal steroid exposure, Apgar score at first and fifth minute, chorioamnionitis, sepsis, mechanical ventilation, periventricular-intraventricular hemorrhage (PIH), PVL, patent ductus arteriosus (PDA) treatment, NEC, retinopathy of prematurity (ROP) surgery, and breastfeeding at discharge. Chorioamnionitis was defined in the presence of at least two of the following signs: maternal increased C reactive protein, leukocytosis, fever above 38°C, malodorous amniotic fluid, and maternal or fetal tachycardia. Sepsis was considered present in the case of a positive blood culture (14).

Three groups of different GAs were identified: group 1 (≤28 weeks' gestation), group 2 (28 weeks + 1 day to 31 weeks + 6 days of gestation), and group 3 (≥32 weeks' gestation). The study was approved by the Ethics Committee of Univerity Hospital of Modena and Reggio Emilia (protocol 205/2015, no 4818). Written consent was obtained from the parents of each neonate enrolled in the study.

### Neurodevelopmental Assessment

In each center, neurodevelopmental follow-up was performed by a multidisciplinary team comprising a neonatologist, a psychologist, a physiotherapist, and a pediatric neurologist. To ensure compliance, parents received appointment reminders by telephone. The survivors were examined neurologically according to the Amiel-Tison neurological assessment (15) with either the Griffiths Mental Developmental Scales (GMDS-R, 1996) (16) or the Bayley Scales of Infant and Toddler Development (BSDI III, 2006) (17), depending on local protocols. The GMDS-R (0–2 years) provides a general development quotient (GQ) of infants' abilities with a mean of 100, a standard deviation (SD) of 12, and five subscale quotients (locomotor; eye and hand coordination; personal and social; hearing and language; cognitive performance), each with a mean of 100 and an SD of 16 (16). The BSDI III provides standardized composite scores for each of the assessed domains (cognitive, fine, and gross motor; receptive and expressive language; adaptive), with a mean of 100 and an SD of 15 (17). For both the GMDS-R and the BSID-III, the cutoff abnormality was two SDs below the normative mean (13, 18). The BSDI III or GMDS-R results were compared among three groups of different GAs.

The infants were also assessed by neuro-functional evaluation according to the International Classification of Disability and Health (ICF-CY) and Neuro-Functional Assessment (NFA ICF-CY) (19, 20). The NFA ICF-CY–based approach has been implemented successfully in routine follow-up programs for preterm infants, providing early identification of neurodevelopmental delay (19–22). Neuro-functional clinical evaluation was performed for cognitive, linguistic, motor, and adaptive function, and then a global NFA ICF-CY score was assigned (Appendix A). All the enrolled patients were also screened for vision (an oculist examination including the fundus oculi) and hearing (brain stem evoked potential). The primary outcome measure was severe functional disability at 2 years of age, corrected for prematurity. Severe functional disability was defined as the presence of at least one among the following outcomes: cerebral palsy (CP), a BSDI III cognitive composite score of <2 SD or a GMDS-R GQ of <2 SD, bilateral blindness (visual acuity < 6/60 in the better eye), bilateral deafness (requiring bilateral hearing aids or unilateral/bilateral cochlear implants), and an NFA ICF-CY > 2. The risk factors for severe functional disability were assessed, and the rate and type of CP were evaluated. CP was defined as a permanent disorder of movement and posture causing activity limitations attributed to non-progressive disturbances that occurred in the developing brain. The classification included spastic CP (monoparesis, hemiparesis, triparesis, tetraparesis, diplegia) and extrapyramidal (dyskinetic) syndromes (23).

### Statistical Analysis

Statistical analyses were performed using Stata Direct Statistical Software version 13 (StataCorp LP, USA). Continuous variables were reported by means and SD or by median and interquartile range (IQR), while categorical variables were reported using frequencies. The groups were compared by χ^2^ analyses for categorical variables and by Kruskal-Wallis tests as non-parametric tests for continuous variables. Several variables (birth weight, GA, site and mode of delivery, ethnicity, gender, multiple gestation, prenatal steroid exposure, Apgar score at first and fifth minute, chorioamnionitis, sepsis, mechanical ventilation, PIH, PDA treatment, NEC, ROP surgery, and breastfeeding at discharge) were evaluated as possible risk factors for functional disability and were presented in the univariate analysis.

The multivariate logistic regression model was built on the basis of a stepwise selection, with entry criteria = 0.05 and stay criteria = 0.1. To assess multicollinearity, the correlation coefficient and variance inflation factor (VIF) were checked. A correlation coefficient level of ≤0.9 and a VIF value of >10 were considered critical values. The best subset of predictors in the multivariate models was determined based on the lowest values of Akaike's information criterion and the Bayesian information criterion (BIC). A *p*-value of <0.05 was considered statistically significant.

## Results

**Figure 1:**
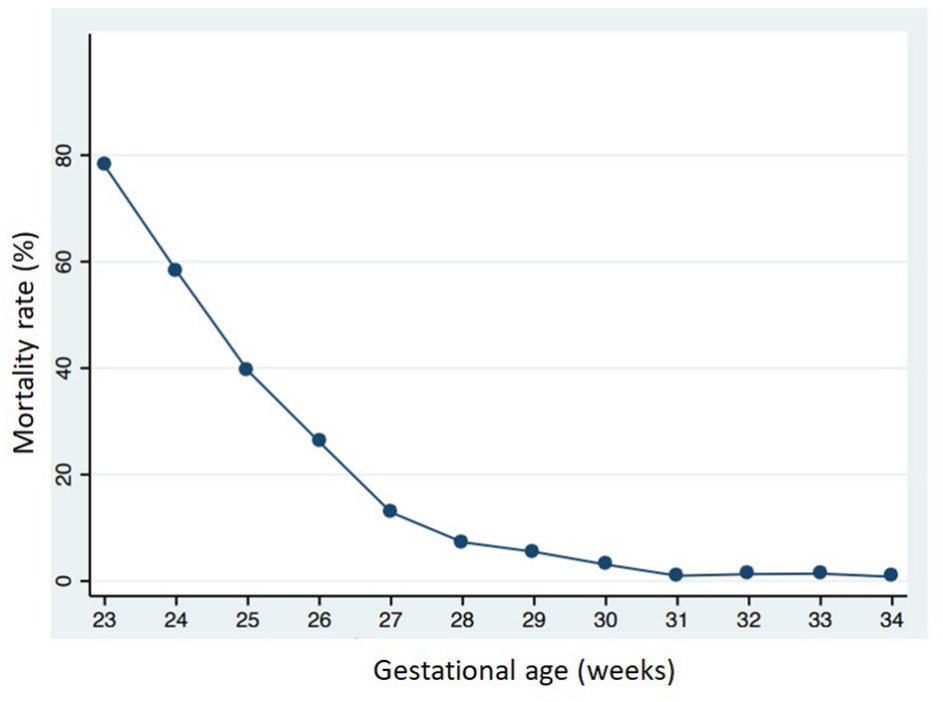
Mortality rate in relation to gestational age at birth.

**Figure 2:**
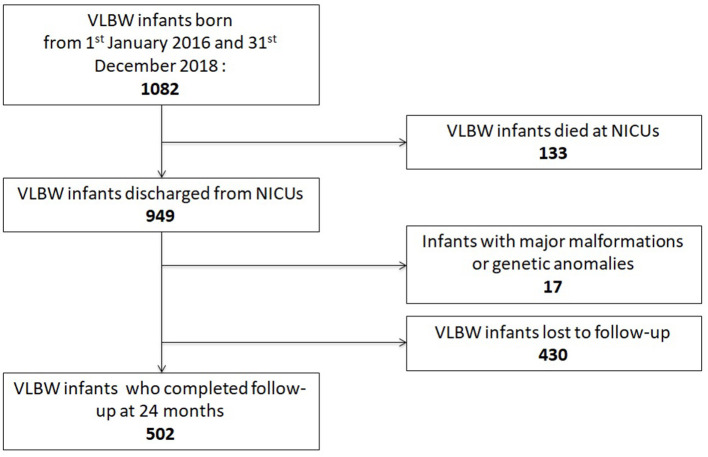
Enrollment flow diagram.

Between 1 January 2016 and 31 December 2018, 1,082 VLBW patients were included in the VON database by participating units. Among them, 133 died (12.3%) within the term-corrected age, before discharge from the hospital: 114/278 (41%) in group 1, 16/249 (6.4%) in group 2, and 3/92 (3.3%) in group 3. Figure 1 shows the mortality rate in relation to GA. Seventeen patients (1.6%) were excluded from the study because of major malformations or genetic anomalies. Among the remaining 932 patients, 502 completed the 24-month neurodevelopmental follow-up (53.9%). Figure 2 shows the enrollment flow diagram. Table 1 compares the characteristics of infants with or without the 24-month neurodevelopmental follow-up. Infants with the 24-month neurodevelopmental follow-up had significantly lower GA at birth; they were more likely to be inborn, delivered after a single pregnancy, and delivered after chorioamnionitis. They were also more likely to be treated for PDA and to be breastfed at discharge from hospital Table 1. Table 2 compares the perinatal characteristics of the three groups, divided according to GA. Patients with a lower GA presented higher rates of chorioamnionitis, sepsis, mechanical ventilation, need for PDA treatment, PIH, NEC, and ROP surgery. In contrast, higher GA infants showed a higher rate of breastfeeding at discharge.

**Table 1:** Characteristics of infants undergoing or not a 24 months neuro-developmental follow-up.

	**Patients with 24 months follow-up** ***N* = 502**	**Patients without 24 months follow-up** ***N* = 430**	**Missing cases**	***p***
Birth weight, median (IQR), g	1,074.5 (870–1,341)	1,270 (1,050–1,415)	–	**<0.001**
Gestational age, median (IQR), wk	29.3 (27.4–31)	30.4 (28.9–32.3)	–	**<0.001**
Outborn, *n* (%)	19 (3.8)	32 (7.4)	–	**0.014**
Ethnicity (Not-Hispanic), *n* (%)	482 (53.2)	424 (46.8)	4	**0.008**
Multiple gestation, *n* (%)	172 (34.3)	194 (45.1)	–	**0.001**
Apgar 1st min, median (IQR)	7 (5–8)	7 (5–8)	–	0.656
Apgar 5th min, median (IQR)	9 (8–9)	9 (8–9)	–	0.115
Cesarean delivery, *n* (%)	94 (18.7)	60 (13.9)	–	0.051
Male gender, *n* (%)	251 (50)	204 (47.4)	–	0.436
Prenatal steroids exposure, *n* (%)	458 (91.9)	387 (90.4)	6	0.406
Chorioamnionitis, *n* (%)	99 (17.8)	58 (13.7)	9	**0.015**
Sepsis, *n* (%)	54 (10.8)	37 (8.6)	–	0.270
Mechanical ventilation, *n* (%)	190 (37.9)	143 (33.3)	–	0.145
Periventricular-intraventricular Hemorrhage, *n* (%)	73 (14.6)	50 (11.7)	4	0.191
Periventricular leukomalacia, *n*(%)	12 (2.4)	7 (1.6)	4	0.412
PDA pharmacological treatment, *n* (%)	130 (25.9)	69 (16.1)	–	**<0.001**
PDA surgical treatment for, *n* (%)	177 (35.3)	98 (22.8)	28	**<0.001**
Necrotizing enterocolitis, *n* (%)	18 (3.6)	23 (5.4)	–	0.191
ROP surgical treatment, *n* (%)	14 (2.8)	11 (2.6)	1	0.833
Human milk feeding at discharge, *n* (%)	342 (68.1)	257 (59.8)	–	**0.008**

*PDA, patent ductus arteriosus; ROP, retinopathy of the prematurity*.

*The values in bold are statistically significant (p < 0.05)*.

**Table 2:** Comparison of neonatal characteristics among different gestational ages groups in patients completing 24 months follow-up^*^.

	**Group 1** **≤28 wks** ***N* = 164**	**Group 2** **28 wks + 1 day to 31 wks + 6 days** ***N* = 249**	**Group 3** **>32 wks** ***N* = 89**	**Missing cases**	***p***
Birth weight, median (IQR), g	825.5 (705–950)	1,175 (980–1,345)	1,405 (1,303–1,480)	–	**<0.001**
Gestational age, median (IQR), wk	26.5 (25.4–27.4)	29.7 (29–30.6)	33.3 (32.3–33.9)	–	**<0.001**
Outborn, *n* (%)	7 (4.3)	10 (4)	2 (2.3)	–	0.698
Ethnicity (Not-Hispanic), *n* (%)	7 (4.3)	9 (3.6)	2 (2.2)	–	0.720
Multiple gestation, *n* (%)	56 (34.2)	85 (34.1)	31 (34.8)		0.992
Apgar 5th min, median (IQR)	8 (7–9)	9 (8–9)	9 (9–10)	–	**<0.001**
Male gender, *n* (%)	89 (35.5)	120 (47.8)	42 (16.7)	–	0.406
Prenatal steroids exposure, *n* (%)	152 (93.8)	226 (91.5)	80 (89.9)	4	0.508
Chorioamnionitis, *n* (%)	64 (39)	32 (12.9)	3 (3.4)	1	**<0.001**
Sepsis, *n* (%)	41 (25)	12 (4.8)	1 (1.1)	–	**<0.001**
Mechanical ventilation, *n* (%)	127 (77.4)	60 (24.1)	3 (3.4)	–	**<0.001**
Periventricular-intraventricular hemorrhage, *n* (%)	45 (27.6)	20 (8.1)	8 (9)	2	**<0.001**
Periventricular leukomalacia, *n* (%)	4 (2.5)	6 (2.4)	2 (2.3)	2	0.994
PDA pharmacological treatment, *n* (%)	87 (53.1)	42 (16.9)	1 (1)	–	**<0.001**
PDA surgical treatment, *n* (%)	28 (17.8)	2 (0.8)	0	15	**<0.001**
Necrotizing enterocolitis, *n* (%)	13 (7.9)	2 (0.8)	0	–	**<0.001**
ROP surgical treatment, *n* (%)	12 (7.3)	2 (0.8)	0	–	**<0.001**
Human milk feeding at discharge, *n* (%)	87 (53.1)	177 (71.1)	78 (87.6)	–	**<0.001**

*Wks, weeks*.

* *Kruskal-Wallis test was used to compare groups*.

*The values in bold are statistically significant (p < 0.05)*.

### Neurodevelopmental Outcome at 24-Month Corrected Age

**Figure 3:**
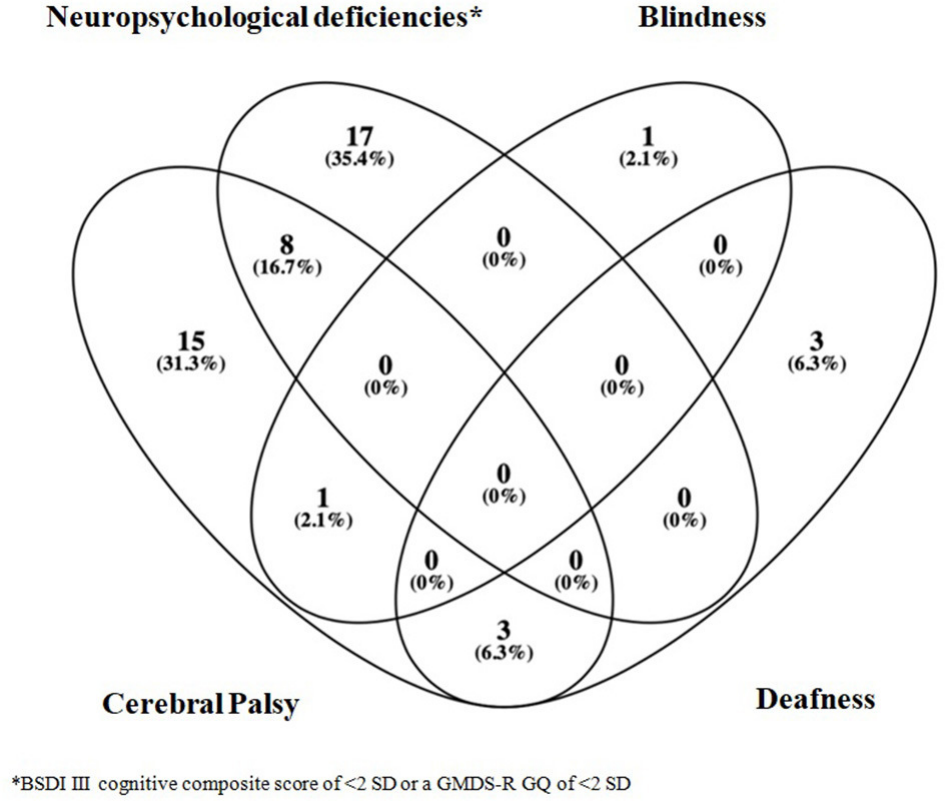
Severe functional disability Venn diagram.

Severe functional disability occurred in 48/502 infants (9.6%). Seventeen of those 48 patients (35.4%) presented a BSDI III cognitive composite score of <2 SD (two cases) or a GMDS-R GQ of <2 SD (15 cases); eight patients presented CP and a BSDI III cognitive composite score of <2 SD (three cases) or a GMDS-R GQ of <2 SD (five cases); 15 patients had CP; one had blindness; three had deafness; one had CP and blindness; and three patients showed CP and deafness (Figure 3). Overall, among the 502 followed-up infants, CP was diagnosed in 27 (5.4%) (monoparesis *n* = 2; hemiparesis *n* = 7; diplegia *n* = 10; tetraparesis *n* = 8), whereas deafness occurred in 6 (1.2%) and blindness in two (0.4%) infants.

Table 3 compares the characteristics of infants with or without severe functional disability at age 24 months. Patients with severe functional disability had lower birth weight and GA, lower fifth-minute Apgar scores, and higher rates of mechanical ventilation, NEC, sepsis, treatment for PDA, and cerebral lesions (assessed by cerebral ultrasound) at discharge from hospital.

**Table 3:** Neonatal characteristics of infants with or without severe functional disability at 24 months.

	**Patients with severe functional disability** ***N* = 48**	**Patients without severe functional disability** ***N* = 454**	**Missing cases**	***p***
Birth weight, median (IQR), g	861.5 (697.5–1,094.5)	1,104 (895–1,360)	–	**<0.001**
Gestational age, median (IQR), wk	26.9 (25.3–29)	29.5 (27.7–31.3)	–	**<0.001**
Outborn, *n* (%)	4 (8.3)	15 (3.3)	–	0.082
Ethnicity (Not-Hispanic), *n* (%)	3 (6.3)	15 (3.3)	–	0.300
Multiple gestation, *n* (%)	13 (27.1)	159 (35.0)	–	0.270
Apgar 5th min, median (IQR)	9 (7–9)	9 (8–9)	–	**0.038**
Male gender, *n* (%)	31 (64.6)	220 (48.5)	–	**0.034**
Prenatal steroids exposure, *n* (%)	44 (91.7)	414 (92)	4	0.936
Chorioamnionitis, *n* (%)	16 (33.3)	83 (18.3)	1	**0.013**
Sepsis, *n* (%)	14 (29.2)	40 (8.8)	–	**<0.001**
Mechanical ventilation, *n* (%)	33 (68.8)	157 (34.6)	–	**<0.001**
Periventricular-intraventricular hemorrhage, *n* (%)	18 (39.1)	55 (12.1)	2	**<0.001**
Periventricular leukomalacia, *n* (%)	7 (15.2)	5 (1.1)	2	**<0.001**
PDA pharmacological treatment, *n* (%)	20 (41.7)	110 (24.2)	–	**0.009**
PDA surgical treatment, *n* (%)	9 (19.2)	21 (4.8)	15	**<0.001**
Necrotizing enterocolitis, *n* (%)	5 (10.4)	13 (2.9)	–	**0.007**
ROP surgical treatment, *n* (%)	8 (16.7)	6 (1.3)	–	**<0.001**
Human milk feeding at discharge, *n* (%)	22 (45.8)	320 (70.8)	–	**<0.001**

*PDA, patent ductus arteriosus; ROP, retinopathy of the prematurity*.

*Missing cases were excluded from the statistical analyses*.

*The values in bold are statistically significant (p < 0.05)*.

**Figure 4:**
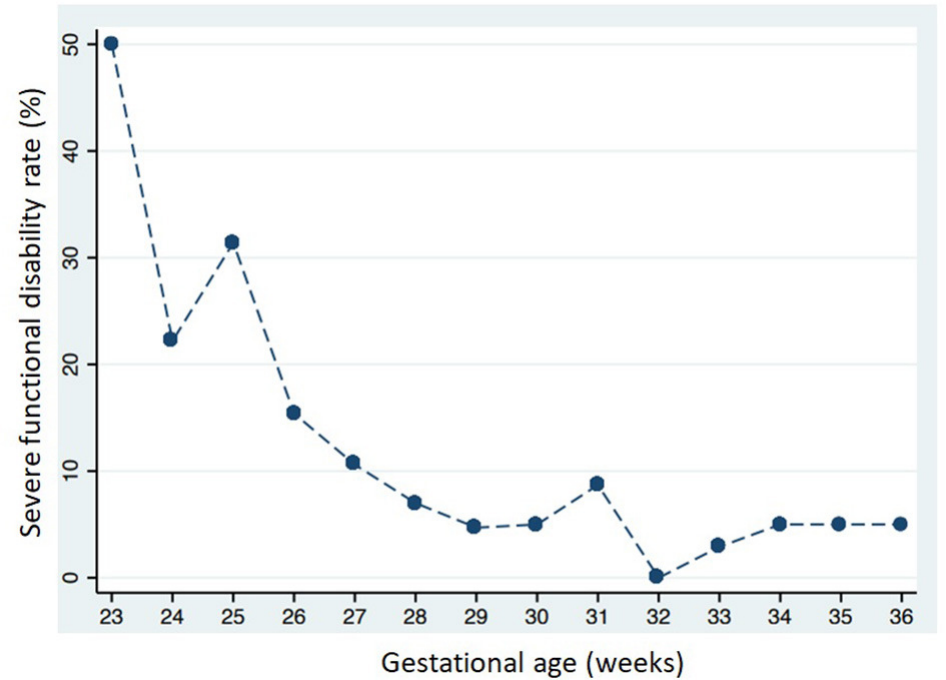
Severe functional disability in relation to gestational age at birth.

Severe functional disability was more common in infants with a lower GA: 31 of 164 (18.9%) in group 1; 15 of 249 (6.0%) in group 2; and two of 89 (2.3%) in group 3 (*p* < 0.001). Figure 4 shows severe functional disability in relation to GA. CP was also more common in infants with a lower GA: 19 of 164 (11.6%) in group 1; six of 249 (2.4%) in group 2; and two of 89 (2.2%) in group 3 (*p* < 0.001). Overall 147 infants (29.3%) were sent to neuromotor rehabilitation during the first 24 months of life, more frequently if they had a lower GA: 83 of 164 (50.6%) in group 1; 58 of 249 (23.3.%) in group 2; and six of 89 (6.7%) in group 3 (*p* < 0.001).

In the univariate regression analysis, several variables were associated with severe functional disability (Table 4). The final multivariate regression model included four variables: GA at birth, male gender, sepsis, and PIH (area under ROC curve: 0.75). In this multivariate regression model, only GA at birth and PIH were associated with severe functional disability (Table 4).

**Table 4:** Association of perinatal data and severe functional disability at 24 month of corrected age.

	**Univariate model**			**Multivariate model**		
	**OR**	**95% CI**	***p***	**OR**	**95% CI**	***p***
Birth weight	0.99	0.99–0.99	**<0.001**			
Gestational age	0.76	0.67–0.85	**<0.001**	0.79	0.67–0.90	**0.001**
Outborn	2.66	0.84–8.36	0.094			
Maternal age	0.95	0.90–0.99	**0.035**			
Ethnicity	0.97	0.55–1.69	0.910			
Multiple gestation	0.69	0.35–1.34	0.273			
APGAR 1st min	0.83	0.72–0.94	**0.006**			
APGAR 5th min	0.85	0.70–1.02	0.087			
Male gender	1.94	1.04–3.60	**0.036**	1.94	0.99–3.77	0.052
Mode of delivery	1.71	0.86–3.37	0.122			
Prenatal steroids exposure	0.96	0.32–2.81	0.936			
Chorioamnionitis	2.23	1.16–4.25	**0.015**			
Sepsis	4.26	2.11–8.59	**<0.001**	2.23	0.99–4.98	0.050
Mechanical ventilation	4.16	2.19–7.89	**<0.001**			
Periventricular-intraventricular hemorrhage	4.66	2.42–8.98	**<0.001**	2.51	1.19–5.26	**0.015**
Periventricular leukomalacia	16.12	4.88–53.15	**<0.001**			
Pharmacological treatment for PDA	2.23	1.21–4.12	**0.010**			
Surgical treatment for PDA	2.37	1.30–4.32	**0.005**			
Necrotizing enterocolitis	3.94	1.34–11.59	**0.013**			
ROP surgical treatment	14.93	4.93–45.16	**<0.001**			
Human milk feeding at discharge	0.35	0.19–0.64	**0.001**			

*PDA, patent ductus arteriosus; ROP, retinopathy of the prematurity*.

*Missing cases were excluded from the statistical analyses*.

*The values in bold are statistically significant (p < 0.05)*.

### Cognitive and Neuropsychological Outcome

Among the 502 infants, 177 were evaluated with the BSDI III and 208 with the GMDS-R (data were missing or incomplete for the remaining 117 patients). The GMDS-R subscales, GMDS-R GQ, and BSDI III composite scores were evaluated and compared among the three GA groups. The GMDS-R GQ, GMDS-R locomotor, and GMDS-R personal and social quotient as well as the BSDI III cognitive composite score differed significantly (Table 5).

**Table 5:** Comparison of GMDS-R quotients or BSD-III scores among 3 groups with different gestational age.

**GMDS-R/BSDI-III mean values^°^**						
		**Group 1** **≤28 wks** **Total *N* = 118**	**Group 2** **28 wks + 1 day to 31 wks + 6 days** **Total *N* = 200**	**Group 3** **>32 wks** **Total *N* = 67**	**Missing cases**	
	**GMDS-R** ***N*** **= 208**	**GMDS-R** ***N*** **= 56**	**GMDS-R** ***N*** **= 106**	**GMDS-R** ***N*** **= 46**		***p*** **^∧^**
Patients evaluated	Global quotient	95.6 ± 14.1	100.7 ± 13.6	101.7 ± 8.9	4	**0.020**
with GMDS-R^*^	Locomotor quotient	96.5 ± 20.3	104.2 ± 20.1	105.3 ± 16.0	2	**0.029**
	Personal and social quotient	98.0 ± 17.2	104.5 ± 16.0	105.3 ± 12.2	1	**0.019**
	Hearing and language quotient	90.4 ± 17.9	96.0 ± 17.0	96.6 ± 13.0	2	0.092
	Eye & Hand Coordination quotient	102.3 ± 14.0	104.6 ± 13.9	104.8 ± 10.2	–	0.424
	Performance quotient	95.2 ± 16.2	99.9 ± 15.7	99.5 ± 14.6	–	0.114
	**BSDI-III** ***N*** **= 177**	**BSDI-III** ***N*** **= 62**	**BSDI-III** ***N*** **= 94**	**BSDI-III** ***N*** **= 21**		
Patients evaluated	Cognitive composite score	90.8 ± 11.7	96.2 ± 7.9	101.2 ± 8.8	–	**<0.001**
with BSDI-III^*^	Motor composite score	88.3 ± 12.8	93.3 ± 7.4	95.5 ± 10.8	9	0.153
	Language composite score	85.9 ± 12.4	90.1 ± 8.5	92 ± 10.3	–	0.095

*Wks, weeks*.

° *Patients with tetraparesis and blindness were excluded*.

* *The number of patients for the different gestational age categories evaluated with BSDI-III was lower than the number of patients evaluated with GMDS-R (p = 0.037). Social-emotional score and adaptive scorewere not reported because of missing data (74/177 and 98/177, respectively)*.

∧ *Kruskal-Wallis test*.

*Missing cases were excluded from the statistical analyses*.

*The values in bold are statistically significant (p < 0.05)*.

### Neuro-Functional Clinical Evaluation (NFA ICF-CY–Based Approach)

**Figure 5:**
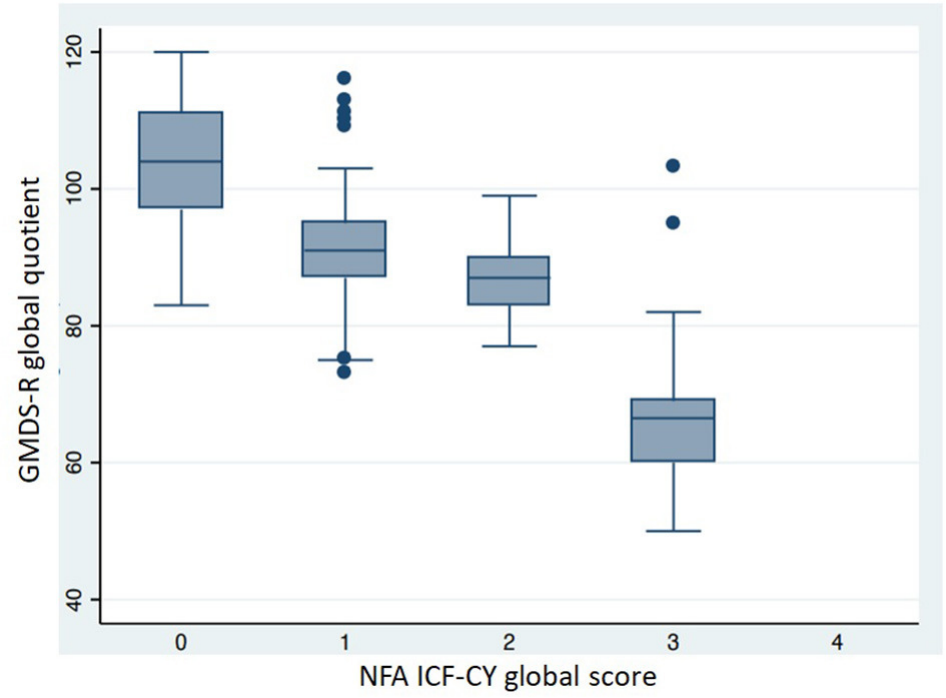
Comparison of GMDS-R global quotient in patients with different NFA ICF-CY score. Patients with tetraparesis and blindness were excluded. Kruskal-Wallis test: *p* < 0.001.

**Figure 6:**
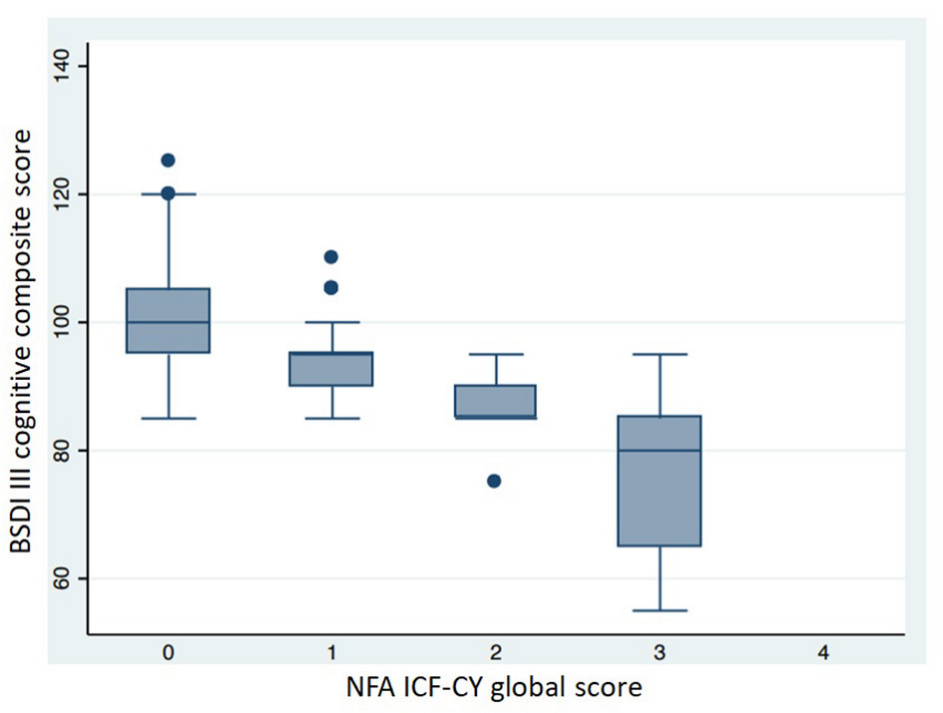
Comparison of BSDI III cognitive composite score in patients with different NFA ICF-CY score. Patients with tetraparesis and blindness were excluded. Kruskal-Wallis test: *p* < 0.001.

Table 6 shows the neuro-functional clinical evaluation. The NFA ICF-CY scores differed significantly between the different GA groups. The GMDS-R GQ and BSDI III cognitive composite scores were lower (*p* < 0.001) in patients with higher NFA ICF-CY scores (Figures 5, 6), distinguishing between patients with major neurodevelopmental anomalies or CP (NFA ICF-CY score > 2) and those with minor anomalies (NFA ICF-CY score ≤ 2).

**Table 6:** Neuro-functional clinical evaluation: results according to different gestational age groups.

**NFA** **ICF-CY**	**Group 1** **≤28 wks** ***N* = 164**	**Group 2** **28 wks + 1 day to 31 wks + 6 days** ***N* = 249**	**≥32 wks** ***N* = 89**	**Missing cases**	***p***
0	69 (42.4)	163 (65.5)	68 (76.4)	1	<0.001
1	53 (32.5)	55 (22.1)	16 (18.0)		
2	10 (6.1)	16 (6.4)	3 (3.4)		
3	21 (12.9)	11 (4.4)	1 (1.1)		
4	10 (6.1)	4 (1.6)	1 (1.1)		

*Wks, weeks*.

## Discussion

In the current study, severe functional disability and CP affected < 10 and 5.4% of survivors, respectively, whereas the literature on the follow-up of preterm neonates reports a higher prevalence of neurodevelopmental disabilities and CP (ranging from 10 to 15%) (24–28). The lower rate of neurodevelopmental disabilities found in the current study probably reflects differences in the study populations. Most previous studies focused on very or extremely preterm newborns, while the current study addressed all VLBW infants, with a GA ranging from 23 to 33 weeks. In our study, the rate of severe functional disability was significantly higher in infants with a lower GA, as those with a ≤28-week GA had eight times higher disability rates than those with a ≥32-week GA. Furthermore, infants with lower GAs had higher rates of CP (11.6% in infants of ≤28 weeks GA) and were more frequently sent to neuromotor rehabilitation. The lower prevalence of severe functional disability and CP in the survivors could also be biased by the number of non-survivors in the groups, but the mortality rate in our study is similar to others in the literature. Specifically, we found a 12.3% overall mortality rate (41% in infants with ≤28 weeks GA), while other studies have reported a 10% mortality rate for under 34 weeks of gestation, 16–18% for under 32 weeks of gestation, and about 40% for extremely preterm infants (29, 30).

Extremely preterm infants are at higher risk of neurodevelopmental disabilities, and preterm birth alone is a significant hindrance to the normal neurodevelopmental trajectory from fetus to adult (31). A healthy intrauterine environment for up to 37–40 weeks of gestation benefits neonatal brain development, while early extra-uterine life interferes with normal brain maturation, increasing the risk of neurological impairment even in the absence of documented cerebral lesions. The early identification of children at risk for subsequent developmental problems may inspire interventions, potentially mitigating the course of otherwise persistent disabilities. In the current study, GA at birth and PIH were significantly associated with severe functional disability. These findings confirm that GA is a main risk factor for poor neurodevelopmental outcomes but also point out the role of cerebral lesions.

Patients with lower GAs showed a higher rate of risk factors for poor neurodevelopmental outcomes, such as chorioamnionitis, sepsis, mechanical ventilation, need for PDA treatment, PIH, NEC, and ROP surgery. In patients with a lower GA, the BSDI III cognitive composite score as far as GQ, locomotor, and personal and social quotients (assessed by GMDS-R) were significantly lower. The BSDI III motor composite score was not significantly different, probably because most of the few patients in the study who were assessed with the BSDI III had a higher GA. Interestingly, the NFA ICF-CY was quite consistent with the BSDI III cognitive composite scores and the GMDS-R GQ. Previous studies have shown the relevance of ICF-CY–based data sets in comparing functioning and disability in children of different ages (29–31). The comprehensive neurodevelopmental assessment based on the NFA ICF-CY approach has been implemented successfully in routine follow-up programs for preterm infants, as it is easy to administer and overcomes data set heterogeneity due to the local protocol of evaluation. The NFA ICF-CY is a useful clinical screening tool for evaluating preterm infants' neurodevelopmental profiles, as it integrates neurological, behavioral, and social items. It enables clinicians to focus on children with suspected developmental delay, who consequently need further assessment or intervention (20–22, 32–34), but the BSID III and GMDS-R remain the gold standard of neurodevelopmental testing in preterm infants. In fact, our study's BSDI III cognitive composite score varies widely among patients with serious anomalies (score 3), who are more roughly assessed by the NFA ICF-CY.

Patients with severe functional disabilities showed documented brain lesions in more than 50% of cases (PIH and PVL in 39 and 15% of cases, respectively). Brain injury is a well-known risk factor for poor neurodevelopmental outcomes. In the current study, cerebral damage was assessed by ultrasounds, and brain lesions were roughly classified according to the VON in PIH and PVL. PIH was significantly associated with severe functional disability, while PVL was less frequent and not associated with a poor outcome, probably due to the small number of children included in the study. Recent studies show that neonatal brain injury, assessed by a standardized magnetic resonance imaging (MRI) scoring system, is associated with short-term neurodevelopmental outcomes, but environmental factors are also important for cognitive development, especially for children with mild neonatal brain injury (35).

The strengths of the Neuroprem study include its prospective design and a very recent enrollment period as well as the inclusion of moderate and very preterm neonates, whose outcomes are reported infrequently. Indeed, the enrollment period (2016–2018) is very close to the present day, reflecting the effect on neonatal outcomes of the most advanced intensive care and support techniques for newborns. By contrast, previous studies included cohorts of preterm infants born before 2015, although the cohorts were larger than ours (1–3, 26, 36).

Our study has some limitations. The first major limitation is that different, although validated, neurodevelopmental scales (the BSDI III, GMDS-R, and NFA ICF-CY) were combined to define disability in our multicenter study because either the BSDI III or the GMDS-R was used depending on the local protocol. The standardization of developmental tests among centers is desirable, but it requires staff training and is costly and time consuming. To overcome data heterogeneity or missing BSDI III/GMDS-R data, the NFA ICF-CY was adopted by all the centers.

A second limitation is incomplete follow-up data for 43% of the neonates, and some might argue that this constitutes selection bias. However, some previous studies report similar dropout rates and suggest an excess of poorly performing children among those not evaluated (37), as children from more disadvantaged families are often lost to follow-up. In the current study, despite the quite high dropout rate, infants who completed follow-up had a lower GA, lower birth weight, and additional risk factors for poor neurodevelopmental outcomes. Therefore, although we lack information regarding the socioeconomic status of the children lost at follow-up, we assume that our results do not underestimate poor outcomes. At the same time, strategies aimed at improving follow-up compliance are desirable in a multicenter context.

A third limitation is that the follow-up did not extend beyond the age of 2 years, and only severe functional disabilities were investigated. Hence, we did not assess mild neurological dysfunction and preschool age performance, which may be impaired in various neuropsychological domains, even in patients without major disabilities. However, the NFA ICF-CY begins to express preliminary data on minor neurodevelopmental anomalies (an NFA ICF-CY score of 2), such as minor motor disorders, minor deficits in cognitive functioning, and regulation difficulties, whose characteristics must be redefined at later ages. Finally, MRI data are lacking, and brain lesions were not described in detail, but these could be areas for future research.

In conclusion, this study provides updated information on the neurodevelopmental outcomes of VLBW infants in a large Italian cohort. The overall rate of neurodevelopmental disabilities was quite lower than in data in the previous literature, and GA remains one of the main risk factors for poor neurodevelopmental outcomes. Interestingly, in this study, CP accounted for just over half of severe functional dysfunction, while the remaining cases presented severe neuropsychological or sensory-neural deficiencies. Neuroprem 2, by providing data on contemporary VLBW outcomes, supports further follow-up programs from a national network perspective. Such networks contribute to promoting access to formal neurodevelopmental evaluation and to timely rehabilitative interventions.

## Data Availability Statement

The original contributions presented in the study are included in the article/Supplementary Material, further inquiries can be directed to the corresponding author/s.

## Ethics Statement

The studies involving human participants were reviewed and approved by the Emilia Romagna Ethics Committee (protocol 205/2015, n 4818). Written informed consent to participate in this study was provided by the participants' legal guardian/next of kin.

## Author Contributions

LLug, FF, and FM contributed to conception and design of the study. MR, NB, ED, MS, AS, and SP organized the database. LB, IG, and LLuc performed the statistical analysis. MP, GA, GG, FS, LC, and OP wrote sections of the manuscript. AB and LI critically revised the manuscript for important intellectual content. Neuroprem Working Group contributed to the follow-up of patients and to data collection. All authors contributed to manuscript revision, read, and approved the submitted version.

## Funding

This study was funded by Pier Franco and Luisa Mariani foundation of Milan, Italy, awarding a grant to LLug.

## Conflict of Interest

The authors declare that the research was conducted in the absence of any commercial or financial relationships that could be construed as a potential conflict of interest.

## Publisher's Note

All claims expressed in this article are solely those of the authors and do not necessarily represent those of their affiliated organizations, or those of the publisher, the editors and the reviewers. Any product that may be evaluated in this article, or claim that may be made by its manufacturer, is not guaranteed or endorsed by the publisher.

## Neuroprem Working Group

Antonella Luglio (Pediatric Post-graduate School University Hospital of Modena and Reggio Emilia, Modena, Italy), Sara Grandi (Neonatal Intensive Care Unit, Infermi Hospital of Rimini, Rimini, Italy), Valentina Fiorini (Neonatal Intensive Care Unit, Azienda USL-IRCCS, Reggio Emilia, Italy), Piero Catenazzi (Neonatal Intensive Care Unit, Maggiore Hospital of Bologna, Bologna, Italy), Elisa Ballardini (Neonatal Intensive Care Unit, Sant'Anna Hospital of Ferrara, Ferrara, Italy), Sabrina Moretti (Neonatal Intensive Care Unit, University Hospital of Parma, Parma, Italy), Daniela Turoli (Italian Neonatal Network, University of Milan, Milan, Italy; Neonatal Intensive Care Unit, Manzoni Hospital of Lecco, Lecco, Italy), Arianna Aceti (Neonatal Intensive Care Unit, Sant'Orsola Malpighi University Hospital of Bologna, Bologna, Italy), Roberto Bellù (Italian Neonatal Network, University of Milan, Milan, Italy; Neonatal Intensive Care Unit, Manzoni Hospital of Lecco, Lecco, Italy), Luca Ori (Neonatal Intensive Care Unit, University Hospital of Modena, Modena, Italy), Silvia Braibanti (Neonatal Intensive Care Unit, Azienda USL-IRCCS, Reggio Emilia, Italy), Alessandra Boncompagni (Neonatal Intensive Care Unit, University Hospital of Modena, Modena, Italy), Caterina Spada (Neonatal Intensive Care Unit, Bufalini Hospital of Cesena, Cesena, Italy), and Lara Valeri (Pediatric Post-graduate School University Hospital of Modena and Reggio Emilia, Modena, Italy).
