# Supplementary material for: Neuroprem 2: An Italian Study of Neurodevelopmental Outcomes of Very Low Birth Weight Infants
Source: Front Pediatr. 2021 Sep 13;9:697100. doi: 10.3389/fped.2021.697100 (PMC8474877; doi:10.3389/fped.2021.697100)
Supplement: Supplementary file 1 [file Table_1.DOC]

**Appendix A: Neuro-functional clinical evaluation NFA ICF-CY**

Mental function:

Normal (1): attentive and curious to the environment and available to play proposals. It organizes itself in the game. Show a preferential relationship with family members. He is able to make transferring games, stack cubes, like to doodle and insert simple geometric shapes. The child explore the environment, indicates to show and request, and shows a desire for autonomy (try to eat, get dressed, and undress). Slightly altered (2): little activation towards the surrounding environment or, on the contrary, continuous changes in the attention focus. He struggles to start a game activity on his own, but lets himself be guided and contained. Little interested in being autonomous, he is still very much helped in dressing and eating. Severely altered (3): little interested in the relationship with people and objects; even when facilitated, the child is not able to regulate himself. Cognitive strategies are poorly organized and rigid. Very dependent on parents

Language:

Normal (1): simple sentences, vocabulary of over 20 words. He makes himself understood. He answers questions concerning himself, even in gestures. He understands what others are saying. Performs small commands.

Slightly altered (2): maximum vocabulary of 20 words does not associate two words. He pauses for a few moments in listening.

Seriously altered (3): the child pronounces less than 10 words, hard to understand simple requests. Look at objects, but cannot name them.

Motor function

Normal (1): independent autonomous walking. He runs, overcomes small obstacles, drag objects while walking. He goes up and down the stairs holding onto a support. He can stick small objects and copy very simple signs.

Slightly altered (2): he walks alone, does not run but trots. The support base is still enlarged and the guard is medium-high. The child tries to climb the stairs, he can't get them down. He holds the pencil to draw but struggles to orient the graphic line.

Severely altered (3): it does not walk or begins to stand up with support. Struggling to overcome obstacles and often stumbles. He holds the objects in his hand, without using them in a functional way, he often throws them

Adaptive function:

Normal (1): normal food functions, regular sleep-wake rhythm. He drinks from the glass and eats with a spoon.

Slightly altered (2): struggling to eat alone, selective about tastes, he starts to taste something. He wakes up at night and struggles to fall asleep.

Severely altered (3): very selective on feeding. He only eats what he wants, he wants to be fed. Very restless sleep, it is hard to calm down after he has woken up, he needs contact with the parent to fall asleep and sometimes even to sleep.

Neuro-functional clinical evaluation score (NFA ICF-CY score)

0. Normality: Normal function, complete movement and interaction patterns.

1. Slight anomalies: They normalize with facilities during the exam and do not involve limitations.

2. Minor anomalies: Anomalies that persist during the examination, disturb the function (motor, postural, adaptive), but do not require adaptations or aids

3. Serious anomalies: Anomalies that significantly disturb the function, the function is only possible with the use of facilitators or devices (eg medium-sized forms of cerebral palsy, with partially preserved functions).

4. Severe pathology: There are serious abnormalities that prevent function, and fixed pathological patterns are evident (eg severe forms of cerebral palsy with no locomotor, manipulative or linguistic function).
